# Hilfsmittelversorgung bei Kindern und Jugendlichen nach der Gesetzesänderung – eine Bestandsaufnahme

**DOI:** 10.1007/s00103-026-04264-0

**Published:** 2026-07-02

**Authors:** Fleming Caje, Peter Borusiak

**Affiliations:** 1 Universität Witten/Herdecke, Witten, Deutschland; 2 Kinderneurologisches Zentrum, LVR-Klinik Bonn, Waldenburger Ring 46, 53119 Bonn, Deutschland

**Keywords:** Hilfsmittel, Familiäre Belastung, Behinderung, Rollstuhl, Teilhabe, Assistive devices, Family burden, Disability, Wheelchair, Participation

## Abstract

**Einleitung:**

Die bedarfsgerechte Versorgung mit Hilfsmitteln wie Rollstühlen, Kommunikationshilfen oder Orthesen hat für Kinder und Jugendliche mit Behinderung im Hinblick auf Teilhabe und Entwicklung eine hohe Bedeutung. Im Februar 2025 wurde im Rahmen des Gesundheitsversorgungsstärkungsgesetzes (GVSG) § 33 Absatz 5c in das Sozialgesetzbuch Fünftes Buch (SGB V) eingefügt, um die Hilfsmittelversorgung bei Verordnungen durch Sozialpädiatrische Zentren (SPZ) und Medizinische Zentren für Erwachsene mit Behinderung (MZEB) zu vereinfachen. Ziel dieser Bestandsaufnahme war die Untersuchung des aktuellen Umsetzungstands und der Auswirkungen der Gesetzesänderung.

**Methoden:**

In einem Mixed-Methods-Ansatz wurden 2 webbasierte Befragungen von Mitarbeitenden in SPZ und betroffenen Eltern durchgeführt. Ergänzend fanden halbstandardisierte Interviews mit verschiedenen Akteuren des Versorgungsprozesses statt: Mitarbeitende aus SPZ, Vertreter*innen der Krankenkassen und Hilfsmittelerbringer.

**Ergebnisse:**

Die Auswirkungen der Gesetzesänderung sind in vielen Bereichen bislang nur begrenzt erkennbar. Es bestehen unterschiedliche Auffassungen zum Vorgehen und zur Notwendigkeit zusätzlicher Prüfungen. Die Belastung der betroffenen Familien bleibt hoch. Die Ergebnisse weisen sowohl auf Unschärfen in der gesetzlichen Regelung als auch auf kommunikationsbedingte Hindernisse bei der Umsetzung hin.

**Diskussion:**

Die aktuelle Situation ist teilweise durch unrealistische Erwartungen, Missverständnisse und Kommunikationshindernisse geprägt. Die Hauptlast tragen weiterhin die Eltern von Kindern mit komplexem Versorgungsbedarf. Eine verbesserte Information und Kommunikation zwischen den beteiligten Akteuren sowie eine Klarstellung der gesetzgeberischen Intention könnten die Umsetzung der Regelung verbessern.

**Zusatzmaterial online:**

Zusätzliche Informationen sind in der Online-Version dieses Artikels (10.1007/s00103-026-04264-0) enthalten.

## Abstract (en)

**Introduction:**

The provision of assistive devices—such as wheelchairs, communication aids, or orthoses—tailored to individual needs is of paramount importance for children and adolescents with disabilities in terms of their participation and development. In February 2025, Section 33 (5c) was added to Book V of the Social Code (SGB V) as part of the Healthcare Strengthening Act (GVSG) to simplify the provision of medical aids when prescribed by social pediatric centers (SPZ) or medical centers for adults with disabilities (MZEB). The aim of this assessment was to examine the current status of implementation and the effects of the legislative change.

**Methods:**

Using a mixed-methods approach, two web-based surveys were conducted among employees at SPZs and affected parents. In addition, in-depth semi-standardized interviews were conducted with various stakeholders in this process: SPZ employees, health insurance representatives, and medical aid providers.

**Results:**

The effects of the legislative change have so far been only partially apparent in many areas. There are differing views on the procedure and the need for additional reviews. The burden on the families affected remains very high. Our survey points to ambiguities in the wording of the legislative change on the one hand and to significant communication-related obstacles on the other.

**Discussion:**

The current situation is partly characterized by false expectations, misunderstandings, and communication barriers. The burden falls largely on the parents of children with complex care needs. Improved information and communication among the stakeholders, as well as a clarification of the legislative intent, could improve the implementation of the regulation.

## Einleitung

Die bedarfsgerechte Versorgung mit Hilfsmitteln wie Rollstühlen, Kommunikationshilfen, Orthesen oder speziellen Sitz- und Lagerungssystemen ist für Kinder und Jugendliche mit Behinderungen eine wichtige Voraussetzung für Teilhabe, Selbstständigkeit und gesundheitliche Entwicklung. Im Februar 2025 erfolgte im Rahmen des Gesundheitsversorgungsstärkungsgesetzes (GVSG) die Einfügung von § 33 Absatz 5c ins Sozialgesetzbuch Fünftes Buch (SGB V), mit der die Hilfsmittelversorgung bei Verordnung aus Sozialpädiatrischen Zentren (SPZ) oder Medizinischen Zentren für Erwachsene mit Behinderung (MZEB) vereinfacht und erleichtert werden sollte.^1^ In der praktischen Umsetzung bestehen jedoch weiterhin Unsicherheiten hinsichtlich der Auslegung der Regelung (siehe Infobox 1). Der Gesetzesänderung vorausgegangen waren konkrete Empfehlungen des Petitionsausschusses des Deutschen Bundestages nach einer Petition einer betroffenen Mutter, wobei die Empfehlungen über die Gesetzesänderung hinausgingen [1]. Neben der Gesetzesänderung wurde bereits zuvor seitens der AG Hilfsmittel der Deutschen Gesellschaft für Sozialpädiatrie und Jugendmedizin (DGSPJ) im September 2023 die Qualifizierte Verordnung (QVO) vorgestellt, die eine teilhabeorientierte und nachvollziehbare Hilfsmittelversorgung ermöglichen sollte [2].

Belastbare und öffentlich einsehbare Zahlen über Auswirkungen der Gesetzesänderung sowie zu den Kosten der Hilfsmittelversorgung von Kindern und Jugendlichen mit Behinderungen liegen bislang nicht vor. Gleichzeitig entsteht bei vielen Betroffenen der Eindruck, dass die mit der Gesetzesänderung verfolgte Vereinfachung bisher nicht ausreichend umgesetzt wird. Dies zeigt sich unter anderem in den Rückmeldungen betroffener Eltern an SPZ, die weiterhin von häufigen Verzögerungen sowie zusätzlichen Prüfungen durch Krankenkassen und den Medizinischen Dienst (MD) berichten. Vor diesem Hintergrund wurden mit der vorliegenden Bestandsaufnahme der aktuelle Stand der Umsetzung der Gesetzesänderung und ihre Auswirkungen untersucht.

## Methoden

Die Erhebung erfolgte in einem deskriptiven Mixed-Methods-Triangulationsdesign. Wir haben 2 webbasierte Befragungen mittels LimeSurvey® (LimeSurvey GmbH, Hamburg, Deutschland) bei Mitarbeitenden in SPZ, die mit der Hilfsmittelversorgung befasst sind, sowie bei Eltern mit betroffenen Kindern und Jugendlichen durchgeführt. Die Fragebögen (siehe Onlinematerial) wurden in einem Konsensverfahren der beteiligten Autoren unter Hinzuziehung der AG Hilfsmittel der Fachgesellschaft erstellt. Anschließend wurden sie in mehreren Pretestläufen unter Beteiligung von Fachleuten und betroffenen Eltern mithilfe von Kommentarfunktionen evaluiert, angepasst und finalisiert. Der Aufruf zur Beteiligung an den Befragungen erfolgte für die Mitarbeitenden in den SPZ über die Kommunikationsplattform „meineDGSPJ“, das Intranet der Fachgesellschaft sowie über einen zusätzlichen E‑Mail-Verteiler mit 135 Mitarbeitenden aus verschiedenen SPZ. Die Eltern wurden über die Social-Media-Kanäle von Reha-KIND informiert. Diese umfassen insgesamt ca. 3000 Abonnenten. Zusätzlich gab es Informationen zur Befragung als Plakate bzw. Aushänge in den SPZ.

Für die qualitativen Einschätzungen wurden je nach Fragestellung 6- bis 7‑stufige Likert-Skalen eingesetzt. Bei den Mitarbeitenden in den SPZ wurden Berufsgruppe, Lage und Größe des SPZ, der Anteil der Arbeitszeit im Bereich Hilfsmittelversorgung, Kenntnisse zur gesetzlichen Regelung und der QVO sowie subjektive Einschätzungen zu diesen Themen erfasst. Darüber hinaus bestand die Möglichkeit von Freitextantworten. Anschließend erfolgte eine Erhebung bzgl. der initial und auch endgültig abgelehnten Hilfsmittel. Zudem wurden subjektive Einschätzungen dazu erfasst, ob Ablehnungen bevorzugt bestimmte Hilfsmittel(gruppen) betreffen und ob Unterschiede im Vorgehen der Krankenkassen wahrgenommen werden. Bei der Befragung der Eltern wurden demografische Daten der Kinder (Altersgruppe, Pflegegrad, Schwerbehindertenausweis, benötigte Hilfsmittel) erfasst und dann eine Einschätzung der aktuellen Situation erbeten. Diese betraf die jeweiligen Verordner, die Hilfsmittelfirmen und die Krankenkassen. Die Antworten wurden online erfasst und aus LimeSurvey® in Excel® exportiert und statistisch ausgewertet. Eine erste Übersicht der qualitativen Antworten wurde dabei mittels MS Copilot® (Microsoft Corporation, Redmond, Washington, USA) generiert und anschließend gewertet.

Zusätzlich zu den webbasierten Befragungen wurden Fokusgruppeninterviews durchgeführt. Zielgruppen waren SPZ-Mitarbeitende, Krankenkassenvertreter*innen, inkl. des MD, Hilfsmittelversorger*innen und Vertreter*innen der Politik. Es wurden zielgruppenspezifische semistrukturierte Interviewleitfäden erstellt und die jeweiligen Beteiligten angefragt. Thematisiert wurden neben persönlicher Rolle und Funktion die wahrgenommenen Veränderungen im Versorgungsprozess seit Inkrafttreten des GVSG im März 2025 (zeitliche Abläufe, Bürokratie und strukturelle Herausforderungen, Rolle des MD etc.), die interprofessionelle Zusammenarbeit und Kommunikation sowie die erlebte Praxis in Bezug auf Genehmigungen und Ablehnungen seitens der Krankenkassen. Ein weiterer Themenkomplex der Interviews betraf die subjektive Bewertung der Qualität der Hilfsmittelversorgung sowie mögliche politische und strukturelle Verbesserungsansätze. Die Mitarbeitenden der AG Hilfsmittel der DGSPJ wurden über das Intranet kontaktiert, Vertreter*innen der Krankenkassen (*n* = 122) sowie die Mitglieder des Gesundheitsausschusses des Deutschen Bundestages per E‑Mail. Erfolgte innerhalb von 14 Tagen keine Rückmeldung, wurde eine Erinnerung versandt.

Die Interviews dauerten jeweils 20–30 min und wurden bis zum Erreichen einer thematischen Sättigung geführt. Mit Einverständnis der Teilnehmenden wurden sie aufgezeichnet und mithilfe der Software Amberscript® (Amberscript Global B.V., Amsterdam, Niederlande) wortgetreu transkribiert. Die Transkripte wurden dann mittels qualitativer Inhaltsanalyse nach Kuckartz ausgewertet [3]. Dabei wurden im Laufe des Forschungsprozesses und auf Grundlage des Materials induktive Hauptkategorien mit Subthemen abgeleitet, anhand welcher das Textmaterial mithilfe der Software MAXQDA® (VERBI – Software. Consult. Sozialforschung. GmbH, Berlin, Deutschland) systematisch codiert wurde. Dieser induktive Ansatz wurde gewählt, da im Rahmen der Bestandsaufnahme zur Hilfsmittelversorgung der Status quo explorativ, ohne die Annahme vorbestehender Theorien, beschrieben und so die Erfahrungen der Prozessteilhabenden unvoreingenommen abgebildet werden sollen.

## Ergebnisse

Die webbasierten Befragungen erfolgten im Zeitraum 09.11.2025–30.11.2025. Die Anfragen zu den Fokusgruppeninterviews wurden Ende Oktober/Anfang November 2025 per E‑Mail verschickt und die Interviews im Zeitraum zwischen dem 01.11. und 08.12.2025 durchgeführt.

### Befragung der Mitarbeitenden von Sozialpädiatrischen Zentren

**Abb. 1:**
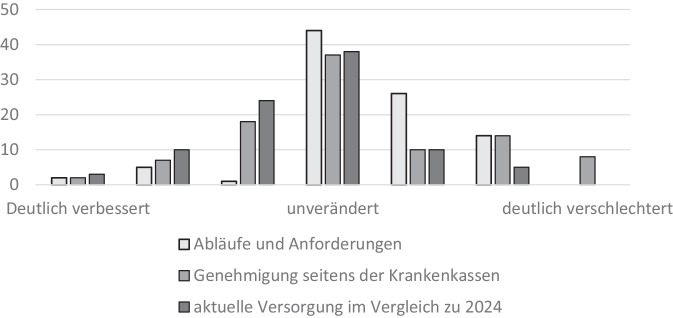
Subjektive Einschätzung der aktuellen Situation im Vergleich zu vor der Gesetzesänderung seitens der Mitarbeitenden aus SPZ (*n* = 99; Angaben in Prozent)

Insgesamt gingen 99 auswertbare Fragebögen ein, darunter Antworten von 42 Ärzt*innen, 31 Physiotherapeut*innen, 8 Logopäd*innen, 4 Ergotherapeut*innen sowie 14 Personen aus weiteren Berufsgruppen oder ohne Angabe der Berufsgruppe. Die subjektiven Einschätzungen zu den allgemeinen Abläufen und Anforderungen der Hilfsmittelversorgung, zum Genehmigungsverhalten der Krankenkassen sowie zur aktuellen Versorgungssituation im Vergleich zum Jahr 2024 sind in Abb. 1 dargestellt. Eine Statistik zur Hilfsmittelversorgung, inkl. Ablehnungen, wurde lediglich von 9 SPZ geführt. Der Anteil der Hilfsmittelverordnungen, die laut den Befragten zunächst abgelehnt oder mit Rückfragen versehen werden, lag im Mittel bei 36,9 % (± 21,5 %, max. 80 %, min. 0,6 %), wobei die Angaben überwiegend auf Schätzungen der Teilnehmenden basieren. Der Prozentsatz der endgültig abgelehnten Hilfsmittel wurde (ebenfalls überwiegend geschätzt) mit 13,1 % angegeben (± 9,9 %, max. 40 %, min. 0 %). Hierbei ließen sich keine klaren regionalen oder SPZ-spezifischen Faktoren (z. B. Größe des SPZ) erkennen.

Auf die Frage, ob bestimmte Hilfsmittel besonders häufig von Ablehnungen betroffen sind, ergab sich ein heterogenes Bild. 14 Befragte verneinten dies. In den übrigen Antworten wurde eine große Bandbreite an Hilfsmitteln genannt, am häufigsten Therapieräder, Rollstühle und Einlagen. Weitere spezifische Hilfsmittel, die häufig abgelehnt wurden, waren Augensteuerungsgeräte, Carbon-Prothesen, GPS-Orthesen, sensomotorische Einlagen, Stehtrainer und Computer für unterstützte Kommunikation, dann auch Mehrfachversorgungen, Zweitversorgungen und spezielle Anforderungen (z. B. Therapierad mit E‑Antrieb, Rollstühle mit Stehfunktion).

Insgesamt gaben 63 von 99 Antwortenden an, Unterschiede in der Genehmigungspraxis der einzelnen Krankenkassen zu bemerken. Als positive Beispiele wurden die AOK (16-mal), die Barmer (9-mal), die BKK (8-mal) und die TK (6-mal) genannt. Bei den negativ hervorgehobenen Kassen wurde am häufigsten die TK (33-mal), die BKKs und die DAK (jeweils 12-mal), die IKK und die AOK (jeweils 10-mal) genannt.

Die qualifizierte Verordnung war den meisten Befragten bekannt (*n* = 86). Von diesen gaben 44 an, sie bei den meisten Hilfsmittelverordnungen zu verwenden, 27 gelegentlich, 6 selten und 12 nie. Positiv hervorgehoben wurden bei der QVO in der offenen Bewertung die strukturierte und übersichtliche Darstellung, die Transparenz und Nachvollziehbarkeit, die Teilhabe- und Alltagsorientierung, die Zielorientierung und individuelle Anpassung, die Standardisierung und auch die rechtliche Verbindlichkeit. Demgegenüber standen als Kritikpunkte der Zeit- und Arbeitsaufwand, die fehlende Akzeptanz und Nutzung durch Krankenkassen, fehlende Digitalisierung, teilweise Länge, Layout und fehlende Flexibilität der Formulare. Zudem wurde eine Verbesserung der Kommunikation zwischen den verschiedenen Akteuren gewünscht.

### Befragung der betroffenen Eltern

Es gingen 161 auswertbare Antworten von Eltern ein. Einige Charakteristika der Kinder sowie die am häufigsten angegebenen Krankenkassen finden sich in der Tab. 1.

**Tab. 1:** Ausgewählte demografische Merkmale sowie die am häufigsten genannten Krankenkassenzugehörigkeiten der Kinder und Jugendlichen (*N* = 161)

Merkmal	Ausprägung	Anzahl (*n*)
Altersgruppen	0–3 Jahre	21
	4–6 Jahre	39
	7–10 Jahre	43
	11–14 Jahre	36
	15–18 Jahre	13
	Keine Angaben	9
Grad der Behinderung	< 70	7
	70	14
	80	27
	90	8
	100	80
	Keine Angaben	25
Pflegegrad	1	0
	2	18
	3	41
	4	38
	5	46
	Keine Angaben	18
Am häufigsten genannte Krankenkassen	TK	34
	AOK (gesamt)	25
	Barmer	11
	BKK (gesamt)	8
	BIG direkt gesund, DAK	Je 4
	IKK, KKH, Knappschaft	Je 2

Bei vielen Kindern und Jugendlichen bestand Bedarf für mehrere Hilfsmittel, eine Auflistung findet sich in Tab. 2. Neben diesen konkret abgefragten Hilfsmitteln wurden weitere Hilfsmittel in Kategorien angegeben: Sitzen & Positionierung (*n* = 24), Mobilität & Fortbewegung (*n* = 19), Pflegebett & Lagerung (*n* = 19), Orthesen/Korsett (*n* = 12), Transport & Auto (*n* = 6), Atmung/Beatmung (*n* = 5), sonstige medizinische Geräte (*n* = 4), Kommunikation (*n* = 3), Sensorik/Alltagshilfen (*n* = 1).

**Tab. 2:** Hilfsmittelbedarf nach Angaben der Eltern (*N* = 161, Mehrfachangaben möglich)

Hilfsmittel	Anzahl
Schienen (Orthesen)	100
Rollstuhl	74
Stehhilfe	53
Reha-Buggy	52
Kommunikation (Talker)	51
Badewannenliege	48
Gehhilfe	47
Einlagen	46
Elektro-Rollstuhl	15

Die meisten Verordnungen erfolgten im SPZ (*n* = 74), teilweise auch bei den niedergelassenen Kinder- und Jugendärzt*innen (*n* = 30), Orthopäd*innen (*n* = 15), in Kindergarten/Schule (*n* = 12) und an weiteren Stellen. Etwa der Hälfte der Eltern war die Gesetzesänderung bekannt (56-mal „ja, ausführlich“ und 29-mal „etwas“), die andere Hälfte hatte weniger (*n* = 19) bzw. keine Kenntnisse (*n* = 50). Insgesamt 99 von 135 antwortenden Eltern gaben an, sich selbst bei der Hilfsmittelversorgung um Organisation oder Nachverfolgung kümmern zu müssen. Die Eltern fühlen sich durch die Hilfsmittelanträge erheblich belastet: Mehr als die Hälfte der Eltern (54,1 %; 73/135) gaben auf der Skala von 1 (= überhaupt nicht) bis 6 (= sehr stark) Werte von 5 oder 6 an. Der Mittelwert lag bei 4,33. Die Belastung führt auch dazu, dass manche Anträge erst gar nicht gestellt werden. Diese Frage wurde von 18 Eltern mit „Ja, häufig“ und von 42 Eltern mit „Manchmal“ beantwortet. Es gingen 135 Antworten auf die Frage ein, ob bei aktuellen Ablehnungen oder Verzögerungen in 2025 ein Gutachten des MD eingeholt wurde. Mit „Ja“ antworteten 67 Eltern, mit „Nein“ 37 („Weiß ich nicht“ oder keine Angabe *n* = 31). Ein Teil der Eltern (*n* = 42) hatte die Krankenkasse auf die neue gesetzliche Regelung hingewiesen. Von diesen berichteten 9 Eltern, dass die Krankenkasse darauf reagiert habe, während 33 angaben, keine Reaktion erhalten zu haben. Die meisten Eltern (75,9 %; 82/108) sehen keine Änderung der Vorgehensweise durch die Gesetzesänderung, 8,3 % (9/108) sehen eher eine Verbesserung, 15,7 % (17/108) eine Verschlechterung. Immerhin wird die Gesamtversorgungssituation im Durchschnitt mit der (Schul‑)Note 2,8 bewertet. Viele konkrete Nöte, aber auch positive Erfahrungen und Verbesserungsvorschläge waren in den Freitextangaben enthalten. Diese sind auszugsweise in Tab. 3 aufgeführt.

**Tab. 3:** Zusammenfassung der Freitextangaben der Eltern bei der webbasierten Befragung

Kategorie	Subkategorie	Kernaussage	Beispielzitat
Strukturelle Versorgungsprobleme	Fehlende interdisziplinäre Anlaufstellen	Familien müssen Versorgung selbst koordinieren, fehlendes SPZ	„Für unseren Sohn gibt es überhaupt keine interdisziplinäre Anlaufstelle …“
	Schlechte Koordination	Akteure stimmen sich nicht ab, kein ganzheitliches Monitoring	„Weder der Kinderarzt noch das Fachkrankenhaus haben hier das ganzheitliche Monitoring im Blick.“
Krankenkassen und MD	Unkenntnis/Ignorieren des Gesetzes	Gesetzesänderung wird nicht angewendet oder bewusst ignoriert	„Die Krankenkassen und der MD wissen teilweise nichts von dem Gesetz, oder wollen es nicht wissen.“
	Taktische Verzögerungen	Verfahren werden langgezogen, MD prüft Wirtschaftlichkeit statt Bedarf	„Es werden Schlupflöcher gesucht … der MD prüfe angeblich nur die Wirtschaftlichkeit.“
	Ablehnungen	Ablehnungen trotz eindeutiger Notwendigkeit, diskriminierende Begründungen	„Er bekommt den Rollstuhl nicht, weil er ihn nicht selbst bedienen kann.“
Zeitliche Verzögerungen	Lange Bewilligungszeit	Bewilligungen dauern Monate bis über ein Jahr	„Prozessdauer einfach zu lange … 6 Monate bis das Hilfsmittel da ist.“
	Lange Lieferzeiten	Hersteller benötigen extreme Lieferzeiten	„9 Monate Wartezeit für ein Therapierad.“
Sanitätshäuser	Überlastung	Rehatechniker kommen nicht hinterher	„Er sagt selbst, dass er nicht hinterherkommt.“
	Schlechte Qualität/Organisation	Unzuverlässige Abläufe, mangelhafte Versorgung	„Man muss dauernd hinterher sein, sonst kommt das Hilfsmittel zu spät.“
	Versorgung aus dem Hilfsmittelpool der Krankenkasse	Hilfsmittel werden nicht rechtzeitig herausgegeben	„Das Sanitätshaus meldet sich einfach nicht zurück.“
Bürokratie	Übermäßige Nachweise	Folgeversorgungen benötigen erneut umfangreiche Begründungen	„Warum muss bei jeder Nachversorgung alles neu ausgestellt werden?“
	Fehlende Digitalisierung	Wunsch nach elektronischen Rezepten und automatischer Übermittlung	„Warum gibt es die Rezepte nicht elektronisch?“
Ungleichheit	Abhängigkeit von Elternressourcen	Versorgung hängt vom Wissen und Einsatz der Eltern ab	„Wir bekommen nur gute Versorgung, weil wir hinterher sind.“
	Unterschiede zwischen Kassen	Versorgungsqualität variiert stark nach Krankenkasse	„Riesige Unterschiede bei den Krankenkassen.“
Belastung der Eltern	Psychische Belastung	Hoher emotionaler Stress durch Kampf um Versorgung	„Es ist unmenschlich, wie man uns behandelt …“
	Zeitliche Belastung	Eltern müssen ständig hinterhertelefonieren, abends Schreiben erledigen	„Ich sitze spätabends noch an Mails an die Krankenkasse.“
Folgen für das Kind	Eingeschränkte Teilhabe	Hilfsmittel fehlen und verhindern soziale Teilhabe	„6 Monate Wartezeit für ein Fahrrad – kein Zustand.“
	Gesundheitliche Risiken	Verspätete Versorgung führt zu Verschlechterungen	„Neue Abdrücke nötig, weil Genehmigung so lange dauerte.“
Positive Beispiele	Gute Krankenkassen	Einige Kassen arbeiten wertschätzend und lösungsorientiert	„Unsere Kasse arbeitet sehr wertschätzend mit uns zusammen.“
	Hilfsmittelversorger als Entlastung	Einige Rehatechniker puffern Systemfehler ab	„Die Hilfsmittelversorger*innen sind die Helden in der Situation.“
Forderungen	Schnellere Prozesse	Bewilligungen und Lieferungen müssen beschleunigt werden	„Von der Beantragung bis zur Genehmigung sollte es schneller gehen.“
	Weniger Bürokratie	Chronische Erkrankungen sollten weniger Neubewertungen benötigen	„Folgeversorgungen sollten nicht wie Neuverordnungen behandelt werden.“
	Mehr Respekt	Eltern wünschen respektvollere, nicht entwürdigende Kommunikation	„Die Erklärungen in den Ablehnungen sind oft unverschämt.“

*MD* Medizinischer Dienst, *SPZ* Sozialpädiatrisches Zentrum

### Vertiefende Interviews

#### Politiker*innen

Es wurden insgesamt 38 Mitglieder des Gesundheitsausschusses mit Bitte um eine Rückmeldung bzw. ein Interview angeschrieben. Zwei Bundestagsabgeordnete haben auf den für die Heil- und Hilfsmittelversorgung zuständigen Abgeordneten ihrer Fraktion verwiesen. Dieser wurde initial bereits kontaktiert, dann nach diesem Hinweis erneut angefragt, ohne dass eine Reaktion bis zum Abschluss der Erhebung vorlag. Zwei weitere Abgeordnete dankten für die Anfrage, gaben an, aktuell wenig beitragen zu können, äußerten jedoch Interesse an den Ergebnissen. Weitere 2 Abgeordnete erklärten ihre Bereitschaft für ein Interview, wobei dies dann ohne uns vorliegende Begründung nicht zustande kam. Von einer Abgeordneten wurde eine schriftliche Anfrage bei der Bundesregierung zu deren Kenntnisstand gestellt, wobei in der Antwort unser initialer Eindruck bestätigt wurde: „Dem Bundesministerium für Gesundheit liegen Hinweise vor, dass bei den Beteiligten Unsicherheiten bezüglich der konkreten Ausgestaltung und der Gültigkeitsvoraussetzungen einer solchen Empfehlung bestehen. Zudem weisen Krankenkassen darauf hin, dass sie häufig nicht erkennen könnten, ob die den Hilfsmittelanträgen zugrundeliegenden Verordnungen oder Empfehlungen aus einem SPZ oder MZEB stammen. Das Bundesministerium für Gesundheit prüft derzeit Möglichkeiten zur Klarstellung, die geeignet sind, die Unsicherheiten der Beteiligten zu beseitigen und die Durchsetzung der vom Gesetzgeber intendierten beschleunigten Versorgung der Betroffenen zu erreichen“ [4].

#### Mitarbeitende aus Sozialpädiatrischen Zentren

In den 3 vertiefenden Interviews mit Mitarbeiter*innen aus SPZ zeigte sich übereinstimmend, dass die Eltern eine zentrale Rolle im Durchlaufen der Prozesse spielen. Dies betrifft die Mitentscheidung bei der Auswahl des Hilfsmittels, dann auch die Antragstellung, die Organisation und Nachverfolgung sowie teilweise auch wiederholte Nachfragen und Widersprüche bei den Krankenkassen. Die Versorgung erfolgt teamgestützt, gemeinsam mit Eltern, Kind, Ärzt*innen und Hilfsmittelanbieter*innen. Die QVO wird von allen genutzt, aber mit unterschiedlichen Erfahrungen. Insgesamt wird die QVO als Verbesserung wahrgenommen, wobei insbesondere hervorgehoben wird, dass medizinische Begründungen und eine Standardisierung erleichtert werden. Weitere Aussagen decken sich mit denjenigen der webbasierten Befragung (bessere Argumentation gegenüber den Kassen, mehr Struktur, aber teilweise umfangreich und zeitintensiv). Von allen übereinstimmend wird der Genehmigungsprozess bei Therapiefahrrädern, Stehtrainern, Therapiestühlen und Kommunikationshilfen als problematisch angesehen. Auch werden besondere Probleme bei Hilfsmitteln für Kinder ohne eindeutig nachvollziehbare (motorische) Einschränkung beschrieben (z. B. bei Autismus-Spektrum-Störung oder kombinierter Entwicklungsstörung). Generell werden seit Anfang 2025 deutlich höhere bürokratische Hürden wahrgenommen. Die Eltern seien zunehmend belastet und müssen häufig neben der Betreuung ihres Kindes bürokratische Aufgaben im Bereich der Hilfsmittelversorgung, insbesondere Organisation und Nachverfolgung, dann auch Nachfragen und Widersprüche bei den Krankenkassen übernehmen. Benannt wird eine „Unplanbarkeit“ aufgrund wechselnder Sachbearbeiter*innen. Zudem gelangen Dokumente nicht zuverlässig vom Kostenträger zum MD. Die Zusammenarbeit mit den Hilfsmittelversorger*innen wird generell als positiv erlebt, allerdings wird auch in diesem Bereich von Überlastung der Mitarbeitenden und lange dauernden Reparaturen berichtet. Gewünscht wurden übereinstimmend eine bessere Kommunikation mit den Krankenkassen, weniger Bürokratie und klarere Abläufe sowie eine kindzentrierte, langfristige Planung statt kurzfristiger Kostenfokussierung. Eine spezifische Idee war ein kindbezogenes Case-Management, inkl. langfristigen Versorgungsplans („roter Faden“ über Jahre).

#### Krankenkassenvertreter*innen

Seit Inkrafttreten der Änderungen im GVSG wurden kaum positive Effekte wahrgenommen. Die Regelungen seien unscharf formuliert, sodass in der Praxis viele Abgrenzungsprobleme entstünden. Insbesondere bei hochwertigen/teuren Versorgungen müsse weiterhin die Wirtschaftlichkeit geprüft werden (§ 12 SGB V).

Als weiteres Hauptproblem wurde u. a. genannt, dass Verordnungen aus SPZ oder MZEB für die Krankenkassen häufig nicht eindeutig erkennbar seien und ihre Identifikation daher eines hohen manuellen Aufwands bedarf. Ferner würden auch Leistungen verordnet, die nicht als Hilfsmittel im Sinne der Richtlinie, sondern eher als Gebrauchsgegenstände einzuordnen seien. Die Zusammenarbeit mit SPZ/MZEB und Leistungserbringern variiere stark, sei teilweise sehr gut, aber generell nicht einheitlich strukturiert. Zudem wurde berichtet, dass Verordnungen häufig unvollständige Angaben enthalten, sodass entstehende Rückfragen den Versorgungsprozess verzögern. Punktuell sei die Zusammenarbeit mit den SPZ auch schwieriger geworden, wenn dem MD Unterlagen mit Hinweis auf § 33 Abs. 5c („medizinische Notwendigkeit wird vermutet“) verweigert würden.

Medizinische Empfehlungen würden bei der Krankenkasse grundsätzlich nicht hinterfragt. Es gebe auch keine MD-Prüfung, sofern der Fall als SPZ/MZEB-Fall erkennbar ist. Geprüft würden vorrangig die Hilfsmitteleigenschaft (z. B. Einhaltung der Hilfsmittelrichtlinie) und die Wirtschaftlichkeit (z. B. Ausschluss von Zubehör ohne Hilfsmittelrelevanz). Änderungen durch Leistungserbringer*innen (z. B. Hinzufügen von nicht notwendigen Komponenten) würden häufig zu Rückfragen oder Teilablehnungen führen. Beschwerden seitens der Versicherten entstünden meist wegen Kommunikationsproblemen, nicht wegen Ablehnungen. Als herausfordernd wird auch die Zuständigkeitsklärung mit anderen Reha-Trägern (Eingliederungshilfe, Jugendhilfe) gesehen. Eine Interviewpartnerin sah keine grundsätzlichen qualitativen Unterschiede zwischen Verordnungen aus SPZ, MZEB und von niedergelassenen Ärzt*innen.

Wir erhielten zudem einzelne schriftliche Rückmeldungen seitens der Krankenkassen, in denen die Kenntnis der Gesetzesänderung bestätigt wurde, gleichzeitig aber auch auf die aktuellen Hindernisse und Unsicherheiten verwiesen wurde. Insbesondere wurden die fehlenden Identifikationsmöglichkeiten von Verordnungen aus einem SPZ oder MZEB benannt.

Zusätzlich kam es am Rande einer Veranstaltung des MD Nordrhein zu einem informellen Austausch. Dabei wurde berichtet, dass die Anfragen nach der Gesetzesänderung konsequent beobachtet worden seien und sich die Zahl der Begutachtungen in diesen Fällen aus Sicht des MD drastisch reduziert habe. Aktuell würden kaum noch Überprüfungen beauftragt. Als zentrale Herausforderungen werden dort strukturelle Probleme gesehen, insbesondere bei der Datenübermittlung, den Datenschutzvorgaben sowie den Speicherfristen. Letztere betragen beispielsweise bei GKV-Daten 5 Jahre, was dazu führe, dass bei Folgeversorgungen von langjährigen Patient*innen häufig erneut sämtliche Unterlagen angefordert und die Entscheidungen neu getroffen werden müssten.

#### Rehatechniker*innen

Die Interviews mit den Leistungserbringer*innen zeigen teils Übereinstimmungen, aber auch Unterschiede in den Erfahrungen mit der Hilfsmittelversorgung. Ein zentrales Thema ist die hohe bürokratische Belastung: Unterschiedliche Anforderungen der Krankenkassen, zusätzliche Formulare und verschiedene Online-Systeme verlangsamen den Genehmigungsprozess. Kostenintensive Versorgungen, insbesondere individuelle Hilfsmittel oder teure Rollstühle, führen zu Verzögerungen. Der MD wird kritisch gesehen, da seine Empfehlungen teilweise als fachlich unzureichend wahrgenommen werden. Gleichzeitig wird berichtet, dass Krankenkassen eigene Entscheidungen mitunter als Entscheidungen des MD darstellen. Persönliche Begutachtungen seien selten, sodass wirtschaftliche und formale Kriterien oft stärker berücksichtigt würden als der tatsächliche Versorgungsbedarf.

Einigkeit besteht in der Bedeutung interdisziplinärer Zusammenarbeit. Versorgungen funktionieren am besten, wenn Ärzt*innen, Therapeut*innen, SPZ-Mitarbeitende und Eltern eng kooperieren. Fehlende Kommunikation könne zu Nachforderungen, Fehlversorgungen oder Verzögerungen führen. Die Gesetzesänderung, die qualitative Verordnungen ermöglicht, wird grundsätzlich positiv bewertet, da sie die Chance bietet, Prozesse auf Basis klarer ärztlicher Empfehlungen zu verbessern. Allerdings sei die Umsetzung uneinheitlich: Manche SPZ seien gut informiert und engagiert, andere weniger, sodass der Erfolg stark vom Einsatz einzelner Personen abhänge.

Die Effekte der Gesetzesänderung werden von den Befragten unterschiedlich bewertet. In einem Interview wird dabei insbesondere die Heterogenität der SPZ hervorgehoben. Zudem werden konkrete bürokratische Hürden beschrieben, darunter verschiedene Statusbögen, unklare Messkriterien sowie der unterschiedliche Umgang mit Foto- und Videodokumentationen. Gleichzeitig wird von erfolgreichen Ansätzen berichtet, etwa der Nutzung der QVO außerhalb eines SPZ, die zu einer Beschleunigung von Genehmigungsverfahren beigetragen habe. Aus der Perspektive der Eltern zeigen sich teilweise enttäuschte Erwartungen, da die angestrebten Verbesserungen noch nicht flächendeckend spürbar seien.

Insgesamt verdeutlichen die Interviews mit den Rehatechniker*innen, dass Bürokratie, heterogene SPZ-Qualität und wirtschaftliche Zwänge zentrale Hemmnisse darstellen. Gleichzeitig seien engagierte Fachkräfte, klare Verantwortlichkeiten und interdisziplinäre Zusammenarbeit entscheidend für eine schnelle, qualitativ hochwertige Versorgung. Die Gesetzesänderung biete eine Chance zur Verbesserung, deren Erfolg jedoch stark vom Wissen und Einsatz der beteiligten Fachkräfte abhängt.

## Diskussion

Wenn die Intention der Gesetzesänderung war, das Genehmigungsverfahren bei der Hilfsmittelversorgung dieser vulnerablen Gruppe zu beschleunigen, so ist dies noch nicht ausreichend gelungen. Vor diesem Hintergrund erscheint auch eine Diskussion darüber sinnvoll, inwiefern eine präzisere gesetzliche Ausgestaltung dazu beitragen könnte, die Intention des Gesetzgebers – eine beschleunigte Versorgung der Betroffenen – zu erreichen. Die Ergebnisse der Elternbefragung und die Antworten aus den SPZ spiegeln die Mühen der Antragsverfahren und die Belastungen der Familien wider.

Nun ist aber nach unseren Ergebnissen die Schuld nicht einseitig zu suchen. Es gibt vielmehr eine Vielzahl an Missverständnissen und v. a. auch Kommunikationsschwierigkeiten. So war den Krankenkassenvertreter*innen die QVO nicht bekannt und diese wurde auch nur von einem Teil der Mitarbeitenden aus den SPZ eingesetzt. Als weiteres Hemmnis wurde von den Krankenkassen benannt, dass häufig nicht erkennbar ist, dass die Verordnung aus einem SPZ oder einem MZEB stammt. Auch liegen nicht alle Verzögerungen an den Krankenkassen, sondern auch fehlende personelle Kapazitäten, z. B. bei den Hilfsmittelfirmen, tragen teilweise dazu bei. Auch konnten wir feststellen, dass häufig eine missverständliche Interpretation der Sachlage erfolgt, wenn davon ausgegangen wird, dass durch die Gesetzesänderung eine automatische Genehmigung vorliegt. Diese Falschinformationen sind auch auf seriösen Fachportalen zu finden und erzeugen falsche Erwartungshaltungen [5]. Erschreckend ist jedoch, dass immerhin 60 Eltern antworteten, dass sie aufgrund der erlebten Situation manche Hilfsmittelanträge gar nicht erst stellen. Allerdings ergaben sich auch gewisse Diskrepanzen in den Antworten. Bei aller Belastung, die sich in den Daten der strukturierten Befragung und den freien Antworttexten zeigt, wurde für die aktuelle Versorgungssituation noch mit einem Durchschnitt von 2,8 die Schulnote „befriedigend“ vergeben.

### Limitationen.

Grundsätzlich müssen einschränkend die Art der Erhebung, die Zahl und auch die Intention der Teilnehmenden berücksichtigt werden. Wir gehen davon aus, dass sich insbesondere Eltern an der Befragung beteiligt haben, bei denen es Schwierigkeiten bei der Hilfsmittelversorgung ihrer Kinder gab. Die einzelnen Interviews mit den verschiedenen Akteuren können auch nicht als repräsentativ für die gesamte Versorgung angesehen werden. Dennoch ergeben sich in der Zusammenschau der Befragungen und Interviews schon einige Gemeinsamkeiten, aus denen sich auch weitere Schlussfolgerungen und Verbesserungen ableiten lassen.

### Ergänzende Handreichung.

Kurz nach Abschluss unserer Erhebung wurden seitens des Aktionsbündnis für bedarfsgerechte Heil- und Hilfsmittelversorgung noch Handreichungen für eine sinnvolle Umsetzung der Hilfsmittelversorgung für die Beteiligten veröffentlicht. „How to Handle § 33, Abs. 5c“ liegt in einer juristisch kommentierten Langform [6] und einer einfach formulierten Kurzform [7] vor. Hiermit soll ein ressourcenschonender und effizienter Informationsaustausch ermöglicht werden. Eine solche Handreichung kann erheblich zur Verbesserung der Abläufe beitragen, da sie die verschiedenen Sichtweisen widerspiegelt.

## Fazit

Bei unserer Erhebung konnten wir viele Missverständnisse und Kommunikationshindernisse feststellen. Den Aussagen (und auch unserem Eindruck nach) sind alle Beteiligten gewillt, die Situation zu verbessern. Übereinstimmend wird von einer sehr hohen Belastung der Familien berichtet. Dabei ist hervorzuheben, dass die Hilfsmittelversorgung lediglich einen Teilaspekt der vielfältigen Herausforderungen darstellt, mit denen Familien von Kindern mit Behinderungen konfrontiert sind.

Aktuell wäre bei Betrachtung der Daten eine Verbesserung des Informationsstandes der Beteiligten und der Kommunikation untereinander förderlich. Hilfreich wären möglicherweise auch webbasierte Lösungen im Sinne einer App, die einerseits eine Nachverfolgung des aktuellen Bearbeitungsstandes, andererseits auch eine statistische Auswertung im Sinne der Versorgungsforschung ermöglichen würde.

Insgesamt sollte übergeordnet noch eine Klarstellung der bestehenden Unsicherheiten seitens des zuständigen Ministeriums erfolgen. In der Antwort der Bundesregierung vom 27.11.2025 wird die Intention der Gesetzesänderung deutlich: „Eine weitergehende Prüfung des Bedarfs durch Krankenkassen oder den medizinischen Dienst (MD) im Rahmen des Genehmigungsverfahrens ist aus Sicht des Gesetzgebers nicht erforderlich und soll angesichts der besonderen Eilbedürftigkeit insbesondere bei der Versorgung von Kindern und Jugendlichen sowie zur Gewährleistung der gesellschaftlichen Teilhabemöglichkeiten unterbleiben.“ Eine Klarstellung dieser Vorgehensweise – wie im gleichen Antwortschreiben formuliert – wäre im Interesse der betroffenen Familien wünschenswert.

Was ist also erforderlich, um die betroffenen Familien zu entlasten?

- Konsequente Nutzung der QVO durch die Mitarbeitenden der SPZ
- Flächendeckende Information der Mitarbeitenden der Krankenkassen über die aktuelle Gesetzeslage
- Eindeutige Kennzeichnung von Verordnungen aus SPZ und MZEB
- Etablierung von Ansprechpartner*innen für die Hilfsmittelversorgung von Kindern und Jugendlichen bei den Krankenkassen
- Vorrangig: eine Klarstellung der Intention der Gesetzesänderung durch das Bundesministerium für Gesundheit

### Infobox Missverständnisse im Genehmigungsverfahren bei Hilfsmittelverordnungen aus Sozialpädiatrischen Zentren (SPZ) und Medizinischen Zentren für Erwachsene mit Behinderung (MZEB)

Grundsätzlich unterliegen alle Verordnungen aus SPZ dem Wirtschaftlichkeitsgebot (§ 12 SGB V). Dieses beinhaltet, dass die Hilfsmittel notwendig, ausreichend, zweckmäßig und wirtschaftlich sein müssen. In der Gesetzesänderung ist nun formuliert, dass die Erforderlichkeit vermutet wird, wenn die Verordnung aus einem SPZ oder MZEB kommt. Dies bedeutet aber nicht, dass die Krankenkasse nicht mehr prüfen darf – auch nicht, dass der Medizinische Dienst (MD) nicht mehr eingeschaltet werden darf. Hierbei gibt es immer wieder Missverständnisse, da einige der Beteiligten der Meinung sind, dass die Überprüfung der Notwendigkeit durch die Krankenkasse entfällt, wenn die Verordnung aus einem SPZ kommt. Die Krankenkassen dürfen weiterhin die formalen Kriterien (z. B. korrekte Diagnose, Indikation, Vertragsprodukt) prüfen, die medizinische Notwendigkeit bewerten, Alternativen oder Zusatzinformationen anfordern und auch den MD einschalten.

## Supplementary Information

ESM 1 Onlinematerial 1: Umfrage-HiMi-SPZ

ESM 2 Onlinematerial 2: Umfrage-HiMi-Eltern

## Data Availability

Die während der vorliegenden Studie erzeugten und/oder analysierten Datensätze sind auf begründete Anfrage bei der Korrespondenzperson erhältlich.

## Footnotes

1 https://www.gesetze-im-internet.de/sgb_5/__33.html, Zugriff 16.06.2026.

## References

[1] https://dserver.bundestag.de/btd/20/085/2008564.pdf (letzter Zugriff am 1. Febr. 2026)

[2] Dreesmann M (2023) Die qualifizierte Verordnung für Hilfsmittel. Kinderärztliche Praxis 94:440–444

[3] Kuckartz U, Rädiker S (2022) Qualitative Inhaltsanalyse: Methoden, Praxis, Computerunterstützung, 5. Aufl. Beltz Juventa, Weinheim, Basel

[4] https://dserver.bundestag.de/btd/21/029/2102979.pdf (letzter Zugriff am 7. Dez. 2025)

[5] https://www.reha-recht.de/infothek/beitrag/artikel/bundestag-beschliesst-aenderungen-in-der-hilfsmittelversorgung (letzter Zugriff am 8. Dez. 2025)

[6] https://rehakind.com/wp-content/uploads/2025/12/Langfassung-How-to-handle-Paragraph-335c-fuer-Fachleute.pdf (letzter Zugriff am 1. Febr. 2026)

[7] https://rehakind.com/wp-content/uploads/2025/12/Hilfsmittel_leichter_bekommen_ok-01.12.25.pdf (letzter Zugriff am 1. Febr. 2026).

