# Supplementary material for: Medical aids for children and adolescents following the legislative change—a status report
Source: Bundesgesundheitsblatt Gesundheitsforschung Gesundheitsschutz. 2026 Jul 2;69(8):945–52. [Article in German] doi: 10.1007/s00103-026-04264-0 (PMC13415268; doi:10.1007/s00103-026-04264-0)
Supplement: Supplementary file 1 — ESM 1 Onlinematerial 1: Umfrage-HiMi-SPZ [file 103_2026_4264_MOESM1_ESM.pdf]

Auswirkungen der Gesetzesänderung 2025 auf die Hilfsmittelversorgung von Kindern und Jugendlichen mit Behinderungen

Hilfsmittelversorgung von Kindern und Jugendlichen – Ihre Erfahrungen nach der Gesetzesänderung 2025

Im Rahmen eines wissenschaftlichen Projekts untersuchen wir die **Auswirkungen der Gesetzesänderung Anfang 2025 (Gesundheitsversorgungsstärkungsgesetz –GVSG)** auf die Hilfsmittelversorgung von **Kindern und Jugendlichen mit Behinderungen**.

Ziel dieser Untersuchung ist es, die aktuelle Versorgungssituation aus der Perspektive der Sozialpädiatrischen Zentren (SPZ) zu erfassen und besser zu verstehen, wie sich die Reform auf Abläufe, Genehmigungsprozesse, Qualität und Zusammenarbeit in der Hilfsmittelversorgung ausgewirkt hat.

Ihre Einschätzung ist dabei von zentraler Bedeutung, da Sie als Expertinnen und Experten die Versorgungsrealität unmittelbar erleben.

Die Befragung dauert etwa **10 Minuten**. Es gibt keine richtigen oder falschen Antworten –im Mittelpunkt steht ausschließlich Ihre persönliche Erfahrung und Wahrnehmung der aktuellen Versorgungssituation. Die Befragung ist bis zum **30. November 2025** geöffnet. Wir freuen uns über Ihre Teilnahme bis zu diesem Datum.

Die Befragung erfolgt **anonym und freiwillig**. Es werden keine personenbezogenen Daten wie Namen, Einrichtungen oder IP-Adressen gespeichert oder weitergegeben. Alle Angaben werden **vertraulich** behandelt und ausschließlich zu wissenschaftlichen Zwecken im Rahmen des Projekts verwendet. Eine Rückverfolgung einzelner Personen oder Institutionen ist ausgeschlossen. Die Datenauswertung erfolgt ausschließlich in aggregierter Form, gemäß den Anforderungen der **Datenschutz-Grundverordnung (DSGVO)**.

Bei Fragen wenden Sie sich bitte an: fleming.caje@uni-wh.de

Fleming Caje Prof. Dr. med. Peter Borusiak

Universität Witten/ Herdecke

In dieser Umfrage sind 21 Fragen enthalten.

Basisdaten

In welcher Berufsgruppe arbeiten Sie im SPZ?

Bitte wählen Sie nur eine der folgenden Antworten aus:

- ◯ Ärztin/ Arzt
- ◯ Physiotherapeut*in
- ◯ Ergotherapeut*in
- ◯ Logopäd*in
- ◯ Psychologin/ Psychologe
- ◯ Sozialdienst
- ◯ Sekretariat/ Verwaltung
- ◯ Sonstiges ______

In welcher Region/ Bundesland liegt Ihr SPZ?

Bitte wählen Sie nur eine der folgenden Antworten aus:

- ◯ Baden-Württemberg
- ◯ Bayern
- ◯ Berlin
- ◯ Brandenburg
- ◯ Bremen/ Niedersachsen
- ◯ Hamburg
- ◯ Hessen
- ◯ Mecklenburg-Vorpommern
- ◯ Nordrhein
- ◯ Westfalen-Lippe
- ◯ Rheinland-Pfalz/ Saarland
- ◯ Sachsen
- ◯ Sachsen-Anhalt
- ◯ Schleswig-Holstein
- ◯ Thüringen

Wie würden Sie die Größe Ihres SPZ einschätzen?

Bitte wählen Sie nur eine der folgenden Antworten aus:

- ◯ klein (bis 2999 Überweisungsscheine/Jahr)
- ◯ mittel (3000–5199 Überweisungsscheine/Jahr)
- ◯ groß (über 5200 Überweisungsscheine/Jahr)
- ◯ weiß ich nicht

Bedeutung der Hilfsmittelversorgung

Welchen Anteil hat die Hilfsmittelversorgung (Beratung, Verordnung, Anpassung) an Ihrer persönlichen Gesamtarbeit?

Bitte wählen Sie nur eine der folgenden Antworten aus:

- ◯ <10%
- ◯ 10-25%
- ◯ 26-40%
- ◯ 41-60%
- ◯ >60%

Wahrnehmung der Gesetzesänderung 2025

Im Februar erfolgte eine Änderung des §33 SGB V. Hierdurch sollte die Hilfsmittelversorgung gerade in Sozialpädiatrischen Zentren vereinfacht werden.

Ist Ihnen diese Gesetzesänderung bekannt?

Bitte wählen Sie nur eine der folgenden Antworten aus:

- ◯ Ja, detailliert
- ◯ Teilweise
- ◯ Nur am Rande
- ◯ Nein

Wie haben sich nach Ihrer persönlichen Erfahrung Abläufe und Anforderungen seit der Gesetzesänderung insgesamt verändert?

Bitte wählen Sie nur eine der folgenden Antworten aus:

- ◯ 1
- ◯ 2
- ◯ 3
- ◯ 4 - unverändert
- ◯ 5
- ◯ 6
- ◯ 7
- ◯ kann ich nicht beurteilen

(1 = deutlich verschlechtert – 7 = deutlich verbessert)

Erleben Sie nach Ihrer persönlichen Erfahrung, dass sich an den Genehmigungen seitens der Krankenkassen etwas verändert hat?

Bitte wählen Sie nur eine der folgenden Antworten aus:

- ◯ 1
- ◯ 2
- ◯ 3
- ◯ 4 - unverändert
- ◯ 5
- ◯ 6
- ◯ 7
- ◯ Kann ich nicht beurteilen

(1= deutlich einfachere Genehmigungen – 7= deutlich mehr Ablehnungen bzw. Überprüfungen durch die Kassen oder den Medizinischen Dienst)

Zögern Sie gelegentlich, ein Hilfsmittel zu verordnen, weil ein sehr hoher bürokratischer Aufwand zu erwarten ist (z. B. Talker, Elektro-Rollstuhl)?

Bitte wählen Sie nur eine der folgenden Antworten aus:

- ◯ Ja, häufig
- ◯ Manchmal
- ◯ Selten
- ◯ Nein

Glauben Sie, dass Eltern mit geringerem sozioökonomischem Status oder Sprachbarrieren besondere Unterstützung benötigen, um Hilfsmittel durchzusetzen?

Bitte wählen Sie nur eine der folgenden Antworten aus:

- ◯ Ja
- ◯ Teilweise
- ◯ Nein

Qualifizierte Verordnung

Ist Ihnen der Begriff „Qualifizierte Verordnung“ (QVO) bekannt?

Bitte wählen Sie nur eine der folgenden Antworten aus:

- ◯ Ja
- ◯ Nein
- ◯ unsicher

Wie häufig nutzen Sie persönlich die Qualifizierte Verordnung?

Bitte wählen Sie nur eine der folgenden Antworten aus:

- ◯ bei den meisten Hilfsmitteln
- ◯ gelegentlich
- ◯ selten
- ◯ nie
- ◯ weiß ich nicht

Wie bewerten Sie die qualifizierte Verordnung im Alltag?

Bitte wählen Sie nur eine der folgenden Antworten aus:

- ◯ 1
- ◯ 2
- ◯ 3
- ◯ 4
- ◯ 5
- ◯ 6

in Schulnoten (1= Sehr gut - 6= Ungenügend)

Was finden Sie an der Qualifizierten Verordnung gut?

Bitte geben Sie Ihre Antwort hier ein:

Was sollte verbessert werden?

Bitte geben Sie Ihre Antwort hier ein:

Genehmigung und Versorgungsrealität

Führen Sie eine Statistik über die verordneten Hilfsmittel?

Bitte wählen Sie nur eine der folgenden Antworten aus:

- ◯ Ja, die folgenden Zahlen sind der Statistik entnommen.
- ◯ Nein, die folgenden Zahlen sind geschätzt.

Wie hoch schätzen Sie den Anteil der Hilfsmittelverordnungen ein, die zunächst abgelehnt oder mit Rückfragen versehen werden?

Bitte wählen Sie nur eine der folgenden Antworten aus:

- ◯ Nicht einschätzbar
- ◯ Sonstiges ______

Wieviel Prozent der Hilfsmittel werden endgültig abgelehnt?

Bitte wählen Sie nur eine der folgenden Antworten aus:

- ◯ Nicht einschätzbar
- ◯ Sonstiges ______

Gibt es bestimmte Hilfsmittel, die nach Ihrer Erfahrung besonders häufig abgelehnt werden?

Bitte wählen Sie nur eine der folgenden Antworten aus:

- ◯ Nein, es gibt kaum spürbare Unterschiede
- ◯ Sonstiges ______

Gibt es dabei Ihrer Erfahrung nach Unterschiede bei einzelnen Krankenkassen?

Bitte wählen Sie nur eine der folgenden Antworten aus:

- ◯ Ja
- ◯ Nein
- ◯ Kann ich nicht beurteilen

Welche Unterschiede bemerken Sie zwischen den einzelnen Kassen?

Bitte geben Sie Ihre Antwort(en) hier ein:

Eher problemlos sind Genehmigungen vor allem bei folgenden Kassen: ______

Schwierig und aufwändig sind Hilfsmittelverordnungen vor allem bei folgenden Kassen: ______

Gesamteinschätzung

Wie bewerten Sie die aktuelle Versorgung im Vergleich zu 2024?

Bitte wählen Sie nur eine der folgenden Antworten aus:

- ◯ 1
- ◯ 2
- ◯ 3
- ◯ 4 - unverändert
- ◯ 5
- ◯ 6
- ◯ 7
- ◯ Kann ich nicht beurteilen

(1= deutlich schlechter – 7= deutlich besser)

Vielen Dank, dass Sie sich die Zeit genommen haben, an dieser Befragung teilzunehmen. Ihre Einschätzungen leisten einen wichtigen Beitrag, um die Hilfsmittelversorgung von Kindern und Jugendlichen in Deutschland nachhaltig zu verbessern!

30.11.2025 – 19:04

Senden Sie Ihre Umfrage ein.

Vielen Dank für die Beantwortung des Fragebogens.
