# Supplementary material for: Medical aids for children and adolescents following the legislative change—a status report
Source: Bundesgesundheitsblatt Gesundheitsforschung Gesundheitsschutz. 2026 Jul 2;69(8):945–52. [Article in German] doi: 10.1007/s00103-026-04264-0 (PMC13415268; doi:10.1007/s00103-026-04264-0)
Supplement: Supplementary file 2 — ESM 2 Onlinematerial 2: Umfrage-HiMi-Eltern [file 103_2026_4264_MOESM2_ESM.pdf]

Auswirkungen der Gesetzesänderung 2025 auf die Hilfsmittelversorgung von Kindern und Jugendlichen mit Behinderungen

Hilfsmittelversorgung von Kindern und Jugendlichen – Ihre Erfahrungen nach der Gesetzesänderung 2025

Im Rahmen eines wissenschaftlichen Projekts untersuchen wir die **Auswirkungen der Gesetzesänderung Anfang 2025 (Gesundheitsversorgungsstärkungsgesetz – GVSG)** auf die Hilfsmittelversorgung von **Kindern und Jugendlichen mit Behinderungen**.

Ziel dieser Untersuchung ist es, die Erfahrungen betroffener Familien zu erfassen und zu verstehen, wie sich die Versorgung seit der Reform in der Praxis verändert hat.

Uns interessiert insbesondere, **wie Sie als Eltern die Hilfsmittelversorgung Ihres Kindes aktuell erleben**, welche Veränderungen Sie wahrnehmen und wo Sie Verbesserungsbedarf sehen.

Ihre Teilnahme ist ein wertvoller Beitrag, um die Versorgungssituation für Kinder und Jugendliche langfristig zu verbessern.

Die Befragung dauert etwa **10-15 Minuten**. Es gibt keine richtigen oder falschen Antworten – im Mittelpunkt steht ausschließlich Ihre persönliche Erfahrung mit der Hilfsmittelversorgung.

Die Befragung ist bis zum **30. November 2025** geöffnet. Wir freuen uns über Ihre Teilnahme bis zu diesem Datum.
Die Befragung erfolgt **anonym und freiwillig**. Es werden keine personenbezogenen Daten wie Namen, Adressen oder IP-Adressen gespeichert oder weitergegeben. Alle Angaben werden **vertraulich** behandelt und ausschließlich **zu wissenschaftlichen Zwecken** im Rahmen des Projekts verwendet. Eine Rückverfolgung einzelner Personen oder Familien ist ausgeschlossen. Die Datenauswertung erfolgt ausschließlich **in aggregierter Form**, gemäß den Anforderungen der **Datenschutz-Grundverordnung (DSGVO)**.

Bei Fragen wenden Sie sich bitte an: fleming.caje@uni-wh.de

Fleming Caje   Prof. Dr. med. Peter Borusiak

Universität Witten/Herdecke

In dieser Umfrage sind 31 Fragen enthalten.

Basisdaten

Es gibt manchmal den Eindruck, dass es bei der Hilfsmittelversorgung auch regionale Unterschiede gibt, daher interessiert uns auch, wo Sie leben.

Bitte wählen Sie eine der folgenden Antworten:
Bitte wählen Sie nur eine der folgenden Antworten aus:

- ○ Baden-Württemberg
- ○ Bayern
- ○ Berlin
- ○ Brandenburg
- ○ Bremen
- ○ Hamburg
- ○ Hessen
- ○ Mecklenburg-Vorpommern
- ○ Niedersachsen
- ○ Nordrhein
- ○ Westfalen-Lippe
- ○ Rheinland-Pfalz
- ○ Saarland
- ○ Sachsen
- ○ Sachsen-Anhalt
- ○ Schleswig-Holstein

Wie alt ist Ihr Kind bei dem eine Hilfsmittelversorgung notwendig ist?

Bitte wählen Sie eine der folgenden Antworten:
Bitte wählen Sie nur eine der folgenden Antworten aus:

- ○ 0–3 Jahre
- ○ 4–6 Jahre
- ○ 7–10 Jahre
- ○ 11–14 Jahre
- ○ 15–18 Jahre

Hat Ihr Kind einen Schwerbehindertenausweis?

Bitte wählen Sie eine der folgenden Antworten:
Bitte wählen Sie nur eine der folgenden Antworten aus:

- ○ Nein
- ○ Sonstiges ______

Zur besseren Einordnung der Situation bitten wir um zusätzliche Angaben zu Ihrem Kind.

Hat Ihr Kind einen Pflegegrad?

Bitte wählen Sie eine der folgenden Antworten:
Bitte wählen Sie nur eine der folgenden Antworten aus:

- ○ Nein
- ○ Sonstiges ______

Hilfsmittel

Welche Art Hilfsmittel benötigt ihr Kind?

Wählen Sie alle zutreffenden Optionen
Bitte wählen Sie alle zutreffenden Antworten aus:

- ☐ Einlagen
- ☐ Schienen (Orthesen)
- ☐ Gehhilfe
- ☐ Stehhilfe
- ☐ Badewannenliege
- ☐ Rollstuhl
- ☐ Elektro-Rollstuhl
- ☐ Talker
- ☐ Reha-Buggy
- ☐ Sonstiges: ______

Wie lange besteht der Hilfsmittelbedarf Ihres Kindes?

Bitte wählen Sie eine der folgenden Antworten:
Bitte wählen Sie nur eine der folgenden Antworten aus:

- ○ unter 1 Jahr
- ○ 1–3 Jahre
- ○ 4–7 Jahre
- ○ länger als 7 Jahre

Wie häufig benötigt Ihr Kind neue oder angepasste Hilfsmittel? *

Bitte wählen Sie eine der folgenden Antworten:
Bitte wählen Sie nur eine der folgenden Antworten aus:

- ○ seltener als einmal jährlich
- ○ etwa einmal jährlich
- ○ mehrmals jährlich
- ○ weiß ich nicht/ keine Angabe

Wo erfolgt die Hilfsmittelversorgung Ihres Kindes überwiegend?

Bitte wählen Sie eine der folgenden Antworten:
Bitte wählen Sie nur eine der folgenden Antworten aus:

- ○ bei der niedergelassenen Kinderärztin
- ○ im Kindergarten bzw. in der Schule
- ○ bei der Physiotherapie (Krankengymnastik), Ergotherapie und/oder Logopädie
- ○ im Sozialpädiatrischen Zentrum
- ○ In einer neuropädiatrischen Ambulanz an der Klinik
- ○ beim Orthopäden
- ○ Sonstiges ______

Wie bewerten Sie den Umgang der Beteiligten mit Ihnen als Familie im Rahmen der Hilfsmittelverordnung? *

Bitte wählen Sie die zutreffende Antwort für jeden Punkt aus:

	1	2	3	4	5	6	Keine Angabe
**Verordner /Arzt**	○	○	○	○	○	○	○
**Therapeut (Physio, Ergo, Logo)**	○	○	○	○	○	○	○
**Sanitätshaus/Reha-Techniker**	○	○	○	○	○	○	○
**Krankenkasse**	○	○	○	○	○	○	○

(1= sehr wertschätzend – 6= sehr wenig wertschätzend)

Gesetzesänderung 2025

Haben Sie von der Gesetzesänderung 2025 im Bereich der Hilfsmittelversorgung gehört?

Bitte wählen Sie eine der folgenden Antworten:
Bitte wählen Sie nur eine der folgenden Antworten aus:

- ○ Ja, ausführlich
- ○ Etwas
- ○ Nur vom Hörensagen
- ○ Nein

Haben Sie bisher aus Erschöpfung oder Sorge vor zu hohem Aufwand einen notwendigen Antrag vermieden, obwohl das Hilfsmittel benötigt wurde?

Bitte wählen Sie eine der folgenden Antworten:
Bitte wählen Sie nur eine der folgenden Antworten aus:

- ○ Ja, häufig
- ○ Manchmal
- ○ Selten
- ○ Nein

Mussten Sie bisher zur Durchsetzung eines Hilfsmittelantrags auch juristische Unterstützung (z. B. Anwalt) in Anspruch nehmen oder hätten diese benötigt, konnten es aber nicht?

Bitte wählen Sie eine der folgenden Antworten:
Bitte wählen Sie nur eine der folgenden Antworten aus:

- ○ Ja, in Anspruch genommen
- ○ Nein, nicht nötig
- ○ Nein, aus z.B. finanziellen Gründen nicht möglich

Antragstellung 2025

Haben Sie <u>in den letzten 9 Monaten</u> ein Hilfsmittel beantragt?

*

Bitte wählen Sie eine der folgenden Antworten:
Bitte wählen Sie nur eine der folgenden Antworten aus:

- ○ Ja
- ○ Nein

Ende der Umfrage

Vielen Dank für Ihre Teilnahme! Da Sie aktuell keine neuen Hilfsmittel beantragt haben, endet die Befragung hier.

Beantworten Sie diese Frage nur, wenn folgende Bedingungen erfüllt sind:
Antwort war 'Nein' bei Frage ' [G04Q27]' (Haben Sie in den letzten 9 Monaten ein Hilfsmittel beantragt?)

Antragstellung 2025

Welches oder welche Hilfsmittel haben Sie <u>in den letzten 9 Monaten</u> beantragt?

*

Beantworten Sie diese Frage nur, wenn folgende Bedingungen erfüllt sind:
Antwort war 'Ja ' bei Frage ' [G04Q27]' (Haben Sie in den letzten 9 Monaten ein Hilfsmittel beantragt?)

Wählen Sie alle zutreffenden Optionen
Bitte wählen Sie alle zutreffenden Antworten aus:

- ☐ Einlagen
- ☐ Schienen (Orthesen)
- ☐ Gehhilfe
- ☐ Stehhilfe
- ☐ Badewannenliege
- ☐ Rollstuhl
- ☐ Elektro-Rollstuhl
- ☐ Talker
- ☐ Reha-Buggy
- ☐ Sonstiges: ______

Welches oder welche der <u>in den letzten 9 Monaten</u> beantragten Hilfsmitteln sind abgelehnt oder stark verzögert worden? *

Beantworten Sie diese Frage nur, wenn folgende Bedingungen erfüllt sind:
Antwort war 'Ja ' bei Frage ' [G04Q27]' (Haben Sie in den letzten 9 Monaten ein Hilfsmittel beantragt?)

Wählen Sie alle zutreffenden Optionen
Bitte wählen Sie alle zutreffenden Antworten aus:

- ☐ Einlagen
- ☐ Schienen (Orthesen)
- ☐ Gehhilfe
- ☐ Stehhilfe
- ☐ Badewannenlige
- ☐ Rollstuhl
- ☐ Elektro-Rollstuhl
- ☐ Talker
- ☐ Reha-Buggy
- ☐ Keines
- ☐ Sonstiges: ______

Wie gut fühlen Sie sich über den <u>aktuellen</u> Ablauf (Antrag, Genehmigung, Anpassung) seitens der verschiedenen Beteiligten informiert?

*

Beantworten Sie diese Frage nur, wenn folgende Bedingungen erfüllt sind:
Antwort war 'Ja ' bei Frage ' [G04Q27]' (Haben Sie in den letzten 9 Monaten ein Hilfsmittel beantragt?)

Bitte wählen Sie die zutreffende Antwort für jeden Punkt aus:

	1	2	3	4	5	6	Keine Angabe
**Verordner**	○	○	○	○	○	○	○
**Sanitätshaus/Rehatechnik**	○	○	○	○	○	○	○
**Krankenkasse**	○	○	○	○	○	○	○

Verwenden Sie hier gerne die bekannten Schulnoten (1= sehr gut bis 6= ungenügend)

Wurde bei <u>aktuellen</u> Ablehnungen oder Verzögerungen ein Gutachten des Medizinischen Dienstes (MD) eingeholt?

*

Beantworten Sie diese Frage nur, wenn folgende Bedingungen erfüllt sind:
Antwort war 'Ja ' bei Frage ' [G04Q27]' (Haben Sie in den letzten 9 Monaten ein Hilfsmittel beantragt?)

Bitte wählen Sie eine der folgenden Antworten:
Bitte wählen Sie nur eine der folgenden Antworten aus:

- ○ Ja
- ○ Nein
- ○ Weiß ich nicht/ Keine Angabe

Wenn eine Ablehnung erfolgte: Haben Sie Widerspruch eingelegt? *

Beantworten Sie diese Frage nur, wenn folgende Bedingungen erfüllt sind:
Antwort war 'Ja ' bei Frage ' [G04Q27]' (Haben Sie in den letzten 9 Monaten ein Hilfsmittel beantragt?)

Bitte wählen Sie eine der folgenden Antworten:
Bitte wählen Sie nur eine der folgenden Antworten aus:

- ○ Ja
- ○ Nein
- ○ Ich habe keine Ablehnung erhalten

Wie nachvollziehbar war die Begründung bei Rückfragen oder Ablehnungen? *

Beantworten Sie diese Frage nur, wenn folgende Bedingungen erfüllt sind:
Antwort war 'Ja ' bei Frage ' [G04Q27]' (Haben Sie in den letzten 9 Monaten ein Hilfsmittel beantragt?)

Bitte wählen Sie eine der folgenden Antworten:
Bitte wählen Sie nur eine der folgenden Antworten aus:

- ○ 1
- ○ 2
- ○ 3
- ○ 4
- ○ 5
- ○ 6
- ○ Kann ich nicht einschätzen/ Keine Angabe

(1= gar nicht nachvollziehbar – 6= sehr nachvollziehbar)

Wurde Ihrer Einschätzung nach bei der Entscheidung Ihrer Krankenkasse primär nach Wirtschaftlichkeit geprüft?

*

Beantworten Sie diese Frage nur, wenn folgende Bedingungen erfüllt sind:
Antwort war 'Ja ' bei Frage ' [G04Q27]' (Haben Sie in den letzten 9 Monaten ein Hilfsmittel beantragt?)

Bitte wählen Sie eine der folgenden Antworten:
Bitte wählen Sie nur eine der folgenden Antworten aus:

- ○ Ja
- ○ Nein
- ○ Weiß ich nicht/ Kann ich nicht einschätzen

Mussten Sie zusätzliche Unterlagen oder ärztliche Begründungen nachreichen? *

Beantworten Sie diese Frage nur, wenn folgende Bedingungen erfüllt sind:
Antwort war 'Ja ' bei Frage ' [G04Q27]' (Haben Sie in den letzten 9 Monaten ein Hilfsmittel beantragt?)

Bitte wählen Sie eine der folgenden Antworten:
Bitte wählen Sie nur eine der folgenden Antworten aus:

- ○ Ja
- ○ Nein
- ○ Kann ich nicht einschätzen/ Keine Angabe

Haben Sie die Krankenkasse mit der neuen Gesetzgebung konfrontiert?

Bitte wählen Sie eine der folgenden Antworten:
Bitte wählen Sie nur eine der folgenden Antworten aus:

- ○ Ja, und die Krankenkasse hat darauf reagiert
- ○ Ja, ohne Reaktion
- ○ Nein

Wie sind Sie krankenversichert?

Bitte wählen Sie eine der folgenden Antworten:
Bitte wählen Sie nur eine der folgenden Antworten aus:

- ○ Private Krankenversicherung
- ○ Sonstiges ______

Mussten Sie sich selbst bei der Hilfsmittelversorgung um Organisation oder Nachverfolgung kümmern? *

Bitte wählen Sie eine der folgenden Antworten:
Bitte wählen Sie nur eine der folgenden Antworten aus:

- ○ Ja
- ○ Nein
- ○ Weiß ich nicht/ Kann ich nicht einschätzen

Wie belastend empfinden Sie <u>in den letzten 9 Monaten</u> den organisatorischen Aufwand rund um Hilfsmittelanträge für sich selber? *

Beantworten Sie diese Frage nur, wenn folgende Bedingungen erfüllt sind:
Antwort war 'Ja ' bei Frage ' [G04Q27]' (Haben Sie in den letzten 9 Monaten ein Hilfsmittel beantragt?)

Bitte wählen Sie eine der folgenden Antworten:
Bitte wählen Sie nur eine der folgenden Antworten aus:

- ○ 1
- ○ 2
- ○ 3
- ○ 4
- ○ 5
- ○ 6
- ○ Kann ich nicht einschätzen/ Keine Angabe

(1= überhaupt nicht – 6= sehr stark)

Abschluss

Haben Sie seit der Gesetzesänderung Veränderungen bemerkt? *

Beantworten Sie diese Frage nur, wenn folgende Bedingungen erfüllt sind:
Antwort war 'Ja ' bei Frage ' [G04Q27]' (Haben Sie in den letzten 9 Monaten ein Hilfsmittel beantragt?)

Bitte wählen Sie eine der folgenden Antworten:
Bitte wählen Sie nur eine der folgenden Antworten aus:

- ○ 1
- ○ 2
- ○ 3
- ○ 4 - keine Veränderung
- ○ 5
- ○ 6
- ○ 7
- ○ Kann ich nicht einschätzen/ Keine Angabe

(1= deutlich verschlechtert – 7= deutlich verbessert)

Was hat sich verbessert?

Beantworten Sie diese Frage nur, wenn folgende Bedingungen erfüllt sind:
Antwort war 'Ja ' bei Frage ' [G04Q27]' (Haben Sie in den letzten 9 Monaten ein Hilfsmittel beantragt?)

Bitte geben Sie Ihre Antwort hier ein:

Was hat sich verschlechtert?

Beantworten Sie diese Frage nur, wenn folgende Bedingungen erfüllt sind:
Antwort war 'Ja ' bei Frage ' [G04Q27]' (Haben Sie in den letzten 9 Monaten ein Hilfsmittel beantragt?)

Bitte geben Sie Ihre Antwort hier ein:

Mit welcher Schulnote bewerten Sie die aktuelle Hilfsmittelversorgung Ihres Kindes insgesamt?

Beantworten Sie diese Frage nur, wenn folgende Bedingungen erfüllt sind:
Antwort war 'Ja ' bei Frage ' [G04Q27]' (Haben Sie in den letzten 9 Monaten ein Hilfsmittel beantragt?)

Bitte wählen Sie eine der folgenden Antworten:
Bitte wählen Sie nur eine der folgenden Antworten aus:

- ○ 1
- ○ 2
- ○ 3
- ○ 4
- ○ 5
- ○ 6

(1= sehr gut – 6= ungenügend)

Gibt es noch etwas, was Sie uns zur Hilfsmittelversorgung mitteilen möchten

Beantworten Sie diese Frage nur, wenn folgende Bedingungen erfüllt sind:
Antwort war 'Ja ' bei Frage ' [G04Q27]' (Haben Sie in den letzten 9 Monaten ein Hilfsmittel beantragt?)

Bitte geben Sie Ihre Antwort hier ein:

Vielen Dank, dass Sie sich die Zeit genommen haben, an dieser Befragung teilzunehmen. Ihre Erfahrungen und Einschätzungen leisten einen wichtigen Beitrag, um die Hilfsmittelversorgung von Kindern und Jugendlichen in Deutschland nachhaltig zu verbessern.

30.11.2025 – 19:03
Senden Sie Ihre Umfrage ein.
Vielen Dank für die Beantwortung des Fragebogens.
